# Pediatric necrotizing pneumonia case report: clinical features, treatment experience, and prospects

**DOI:** 10.3389/fmed.2025.1619981

**Published:** 2025-09-17

**Authors:** Jingli Zhang, Yingqian Zhang, Chenchu Duan, Tengteng Zhang, Yujing Zhao, Lina Zhen

**Affiliations:** Third Department of Respiratory, Hebei Children's Hospital, Innovation Capacity Improvement Project of Hebei Provincial Pediatric Health and Clinical Research Centre, Shijiazhuang, China

**Keywords:** necrotizing pneumonia, pediatric patients, clinical manifestations, laboratory findings, imaging characteristics, treatment strategies, risk factors, case report

## Abstract

Necrotizing pneumonia (NP) is a rare but severe complication of community-acquired pneumonia in children, often mimicking Congenital Pulmonary Airway Malformation and presenting significant diagnostic and therapeutic challenges. We report the case of a 2-year-old boy with a prolonged and complex course of NP, initially suspected to have Congenital Pulmonary Airway Malformation. The patient presented with persistent fever, cough, and dyspnea, accompanied by co-infections with *Streptococcus pneumoniae*, influenza A (H3N2), human metapneumovirus, and *Mycoplasma pneumoniae*. Imaging revealed progressive lung consolidation evolving into cavitary lesions and pneumothorax. Laboratory tests showed leukopenia with a left shift, neutrophil toxic changes, and reduced CD3^+^, CD8^+^ T cells, NK cells, and IgG levels, suggestive of secondary immunosuppression, although an inborn error of immunity could not be fully excluded. Given the patient’s age and ongoing lung development, conservative management with targeted anti-infective therapy and supportive care was prioritized over surgical resection. By 9 months of follow-up, the cavitary lesions had almost completely resolved, whereas persistent bronchiectasis was still evident at 17 months. The child remained clinically well without recurrent infections. This case underscores that leukopenia does not rule out bacterial infection, particularly in the presence of left shift and neutrophil toxic changes. It highlights the importance of immunologic evaluation, vaccination against pneumococcus and influenza, long-term imaging follow-up, and cautious consideration of surgery in young children with NP.

## Introduction

Necrotizing pneumonia is a rare but severe lung infection characterized by rapid necrosis of lung tissue, often accompanied by the development of cavities. Clinically, patients typically present with persistent fever, breathing difficulties, coughing, chest pain, and other severe symptoms, with rapid deterioration of the condition that may lead to compromised respiratory function and severe complications (1). The pathogenesis of this condition involves inflammatory responses triggered by infection, the impact of bacterial virulence factors, and the host’s immune reactions, culminating in the destruction and necrosis of lung tissues (2, 3).

Within the medical domain, research on necrotizing pneumonia in pediatric populations has been a focal point of interest. Despite its relatively low incidence, the condition often presents with severe symptoms, posing significant challenges for effective treatment. Understanding the clinical features, pathogenesis, and diagnostic and therapeutic strategies of necrotizing pneumonia in children is paramount for enhancing the survival rates and quality of life of affected young patients. Thus, in-depth investigations into this disease carry substantial clinical significance and scientific value.

This study aims to deepen the understanding of necrotizing pneumonia in children by conducting a detailed analysis of a case involving a 2-year-old boy afflicted by this condition, elucidating its clinical presentation, laboratory outcomes, and radiological features. By integrating cutting-edge research findings with clinical expertise, this study seeks to provide a profound understanding of the diagnosis and treatment of necrotizing pneumonia, offering valuable guidance for managing similar cases in the future. Through this research endeavor, we aspire to contribute to a comprehensive comprehension of necrotizing pneumonia management in children, foster further advancements in related research fields, and contribute to the enhancement of diagnostic and therapeutic practices for pediatric respiratory illnesses.

## Presentation

**Figure 1:**
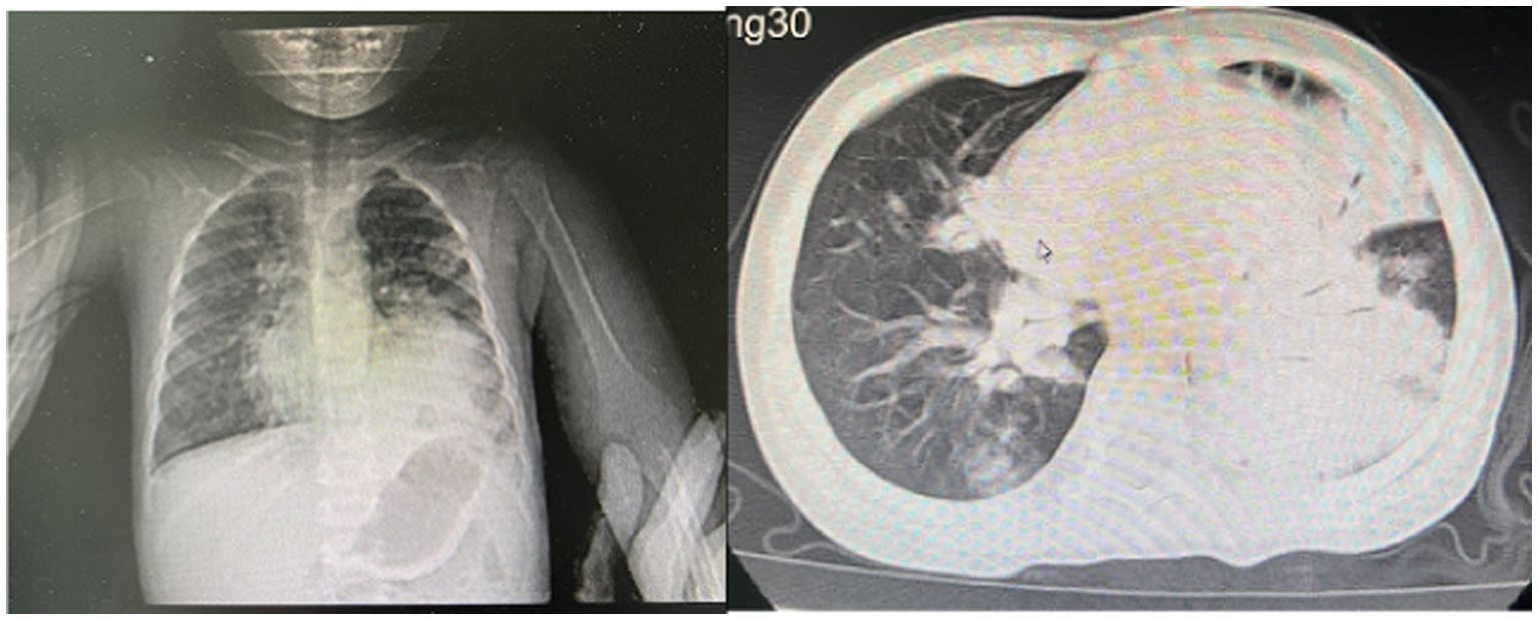
Chest CT scan on the 6th day of the illness.

A 2-year-old male patient presented with a 7-day history of paroxysmal cough with sputum and rhinorrhea, followed by 4 days of fever (peak temperature 39.7 °C). No wheezing or shortness of breath was noted. Prior outpatient treatment with cefuroxime, azithromycin, and methylprednisolone sodium succinate yielded no improvement. Initial blood tests revealed white blood cell count (WBC) 3.7 × 10^9/L, lymphocytes (LY) 26%, neutrophils (NEUT) 67%, hemoglobin (HGB) 120 g/L, platelet count (PLT) 243 × 10^9/L, and C-reactive protein (CRP) 148.24 mg/L. Chest computed tomography (CT) (Figure 1) indicated pneumonia with lung consolidation, prompting hospital admission for severe pneumonia.

On admission, physical examination showed a heart rate of 130 beats/min, respiratory rate of 44 breaths/min, and normal blood pressure. The child was alert, with mild intercostal retractions. Auscultation revealed decreased breath sounds in the left lung, moist rales, and slight wheezing. The abdomen was soft, with the liver palpable 3 cm below the costal margin. Past medical and developmental history was unremarkable, with healthy parents and no family history of genetic disorders. The child had received routine immunizations, including Bacillus Calmette–Guérin (BCG), hepatitis B vaccine, poliomyelitis vaccine, acellular diphtheria–tetanus–pertussis vaccine (DTaP), measles–mumps–rubella (MMR) vaccine, live attenuated Japanese encephalitis vaccine, group A meningococcal polysaccharide vaccine, and live attenuated hepatitis A vaccine. The patient had not received influenza or pneumococcal vaccinations.

Treatment was promptly initiated with intravenous cefotaxime sulbactam sodium (150 mg/kg/day for 10 days) to address the underlying bacterial infection, supported by elevated C-reactive protein (CRP, 148.24 mg/L) and chest CT findings of lung consolidation. Concurrently, intravenous methylprednisolone sodium succinate (2 mg/kg/day for 3 days) was administered to manage severe inflammation. The rationale for corticosteroid therapy was based on clinical and laboratory evidence of a hyperinflammatory state, characterized by persistent high fever (peak 39.7 °C), systemic inflammatory response symptoms (tachycardia at 130 beats/min and tachypnea at 44 breaths/min), and markedly elevated CRP. This approach aimed to mitigate excessive inflammation, reduce the risk of further lung tissue damage, and improve clinical outcomes in the context of poor response to prior outpatient antibiotic therapy.

On day 7 since symptom onset, blood tests showed WBC 3 × 10^9/L, NEUT 74.4% with toxic granulation and vacuolation, CRP 172.1 mg/L, erythrocyte sedimentation rate (ESR) 83 mm/h, procalcitonin (PCT) 11.75 ng/mL, interleukin (IL)-8425.35 pg./mL, IL-6 48.03 pg./mL, IL-10 7.15 pg./mL, D-dimer 1.19 mg/L, lactate dehydrogenase (LDH) 470 U/L, hydroxybutyrate dehydrogenase (HBDH) 343 U/L, total protein 49.7 g/L, albumin 31.1 g/L, prealbumin <70 g/L, and low calcium 2.15 mmol/L, normal 2.2–2.7 mmol/L; ionized calcium 0.96 mmol/L, normal 1.1–1.34 mmol/L.

Lymphocyte immunophenotyping showed reduced T-cell and natural killer (NK) cell populations with an expanded B-cell population: CD3 + 36.59% (normal 53.88–72.87%), CD3 + CD4 + 23.67% (normal 24.08–42.52%), CD3 + CD8 + 11.95% (normal 19.00–32.51%), CD19 + 57.46% (normal 13.23–26.39%), CD56 + CD16 + 2.97% (normal 7.21–20.90%), CD3 + CD4-CD8–0.97%, and CD3 + CD4 + CD8 + 0%. Absolute lymphocyte subset counts were not performed due to limitations in the testing protocol. Serum immunoglobulin levels were: IgG 3.56 g/L (reference range 4.53–9.16 g/L), IgA 0.32 g/L (reference range 0.2–1.0 g/L), IgM 0.81 g/L (reference range 0.19–1.46 g/L). IgE levels were not assessed. The patient had no prior history of recurrent infections or family history of severe infections or autoimmune diseases.

**Figure 2:**
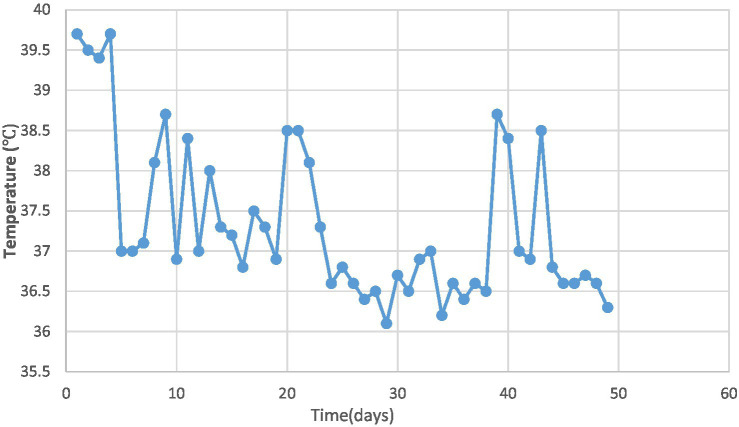
Trend of temperature changes in the pediatric patient.

On day 9, bronchoalveolar lavage revealed congested bronchial mucosa with minimal white secretion. Microbiological testing via polymerase chain reaction (PCR) on bronchoalveolar lavage confirmed *Streptococcus pneumoniae*, human metapneumovirus (hMPV), and influenza A virus subtype H3N2. Serotyping for *S. pneumoniae* was not performed. Peramivir (10 mg/kg/day intravenously for 2 days) was added to the regimen to address influenza A (H3N2). Repeat blood tests showed WBC 19.5 × 10^9/L, NEUT 65.7% with nuclear left shift, and CRP 5.48 mg/L. Despite treatment, intermittent fever (Figure 2), cough, and shortness of breath persisted.

On day 15, chest CT revealed multiple irregularly shaped, thick-walled cavitary lesions in the lower and lingular lobes of the left lung, suggestive of pulmonary necrosis and lung abscess, with Congenital Pulmonary Airway Malformation (CPAM) not excluded (Table 1). Additional diagnoses included necrotizing pneumonia and possible lung abscess.

**Table 1:** Key imaging and clinical differences among necrotizing pneumonia, lung abscess, and CPAM.

Feature	Necrotizing pneumonia	Lung abscess	CPAM (congenital pulmonary airway malformation)
Onset	Acute, post-infectious	Acute/subacute, often after aspiration or severe pneumonia	Congenital, may be asymptomatic until infection
CT Findings	Multiple thin/irregular-walled cavities within consolidated lung	Single or few thick-walled cavities, often with air–fluid levels	Multicystic lesion with thin walls, usually without surrounding consolidation unless infected
Distribution	Infected lobe or segment, often extensive destruction	Localized, usually one lobe	Localized, usually single lobe (commonly lower lobes)
Evolution	Dynamic changes: cavitation, pneumothorax, partial resolution over time	Lesion remains persistent, does not resolve with antibiotics; may require drainage or surgical intervention; potential for chronicity or recurrence if untreated	Stable cystic structure unless infected; may show infection-related changes (e.g., consolidation, air–fluid levels) with potential for resolution or persistence of underlying malformation

On day 16, antibiotics were escalated to linezolid (30 mg/kg/day intravenously for 10 days) due to persistent fever and worsening lung CT findings (pulmonary necrosis and abscess) after 10 days of cefotaxime sulbactam sodium treatment, suggesting difficult infection control and potential drug-resistant *Streptococcus pneumoniae*. By day 20, increased cough and paroxysmal dyspnea were noted, with chest CT showing enlarged cavities, extensive pulmonary destruction, and rightward mediastinal shift. Treatment continued with antibiotics, and surgical resection of necrotic lung tissue was considered. Blood tests on day 13 showed peak WBC of 28.2 × 10^9/L and platelets of 595 × 10^9/L; by day 22, WBC normalized.

**Figure 3:**
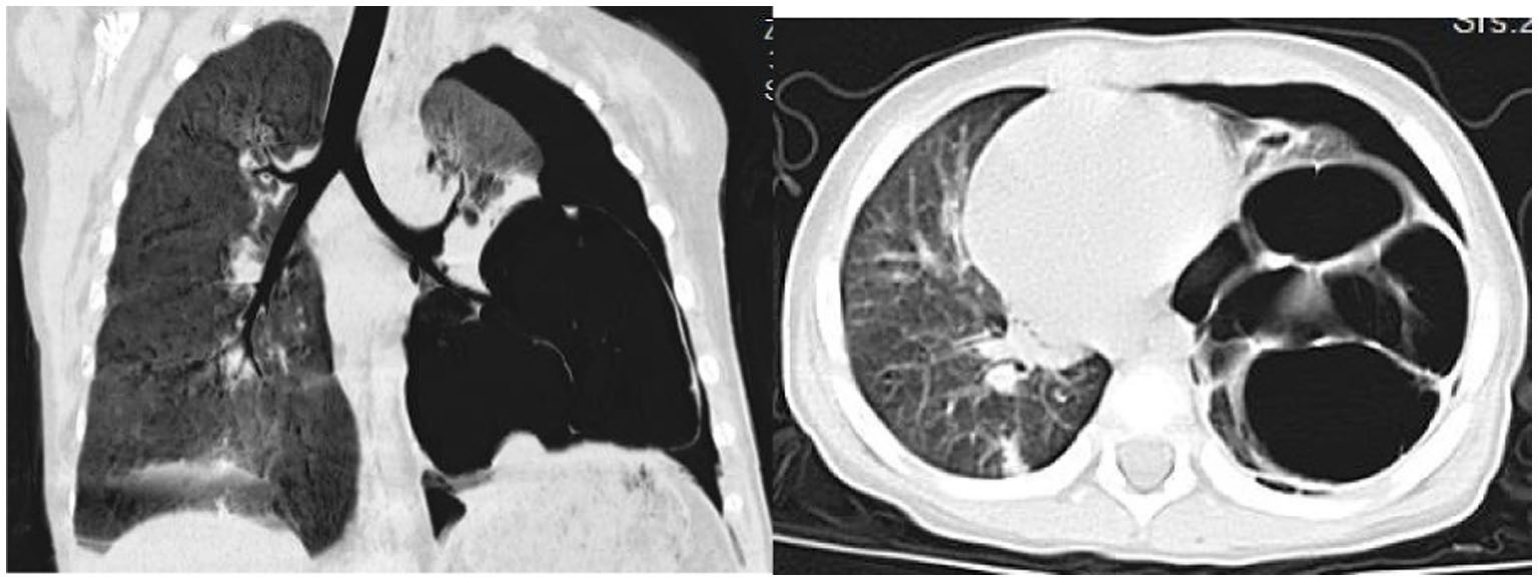
Chest CT scan on the 32th day of the illness.

On day 32, the patient experienced worsened cough and developed respiratory distress. Chest CT (Figure 3) revealed left-sided pneumothorax with 40–50% lung compression, managed with thoracic drainage. Due to significant necrotic lung tissue and potential recurrent pneumothorax, surgical resection was advised but declined by the family. On day 41, recurrent fever prompted testing, revealing *Mycoplasma pneumoniae* via antigen detection on nasopharyngeal swabs using the colloidal gold method, treated with azithromycin (10 mg/kg/day intravenously for 3 days).

**Figure 4:**
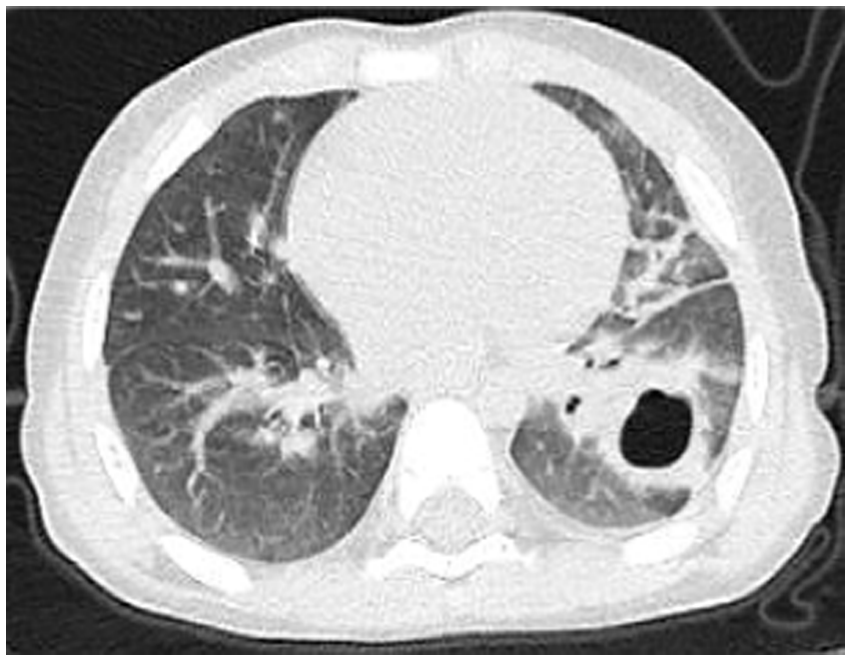
Chest CT scan on the 67th day of the illness.

**Figure 5:**
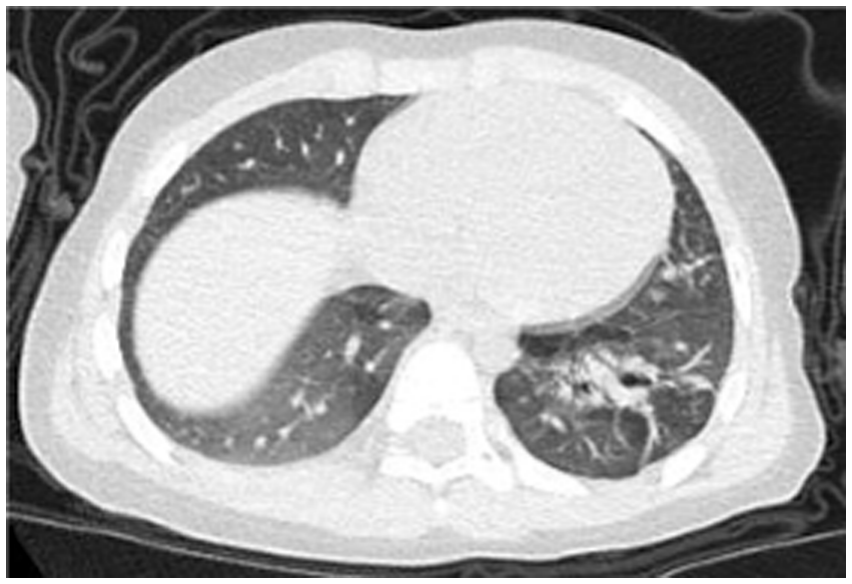
Chest CT scan at the 17th month of the illness.

Post-treatment, the patient’s temperature normalized, with occasional cough but no dyspnea or respiratory distress. The patient was discharged. Follow-up chest CT on day 67 (Figure 4) showed reduced cavitary lesion size and number, with surrounding consolidations. By 9 months post-onset, further reduction in cavity size, consolidations, fibrous strands, and localized bronchiectasis was noted. By 17 months (Figure 5), only minimal residual cavitary lesions, consolidations, and localized bronchiectasis were observed, without further significant changes compared with the 9-month follow-up. The patient’s clinical Event, Diagnostic Findings, and key interventions are summarized in Table 2.

**Table 2:** Key clinical events timeline.

Time (day from symptom onset)	Clinical events & findings	Interventions	Outcomes
Day 0–3	Paroxysmal cough with sputum and rhinorrhea	Outpatient cefuroxime, azithromycin, methylprednisolone	No improvement
Day 4–6	Persistent fever (peak 39.7 °C), tachypnea (RR 44/min), lung consolidation on chest CT	Hospital admission; IV cefotaxime–sulbactam; IV methylprednisolone (3 days)	Mild relief, but fever persisted
Day 7	WBC 3.0 × 10^9/L with toxic neutrophils; CRP 172 mg/L.	Supportive care	Inflammation markers rising
Day 9	Bronchoscopy: congested mucosa, little secretion. BALF PCR positive for *S. pneumoniae*, influenza A (H3N2), and hMPV	Added IV peramivir (2 days)	WBC rebound 19.5 × 10^9/L; intermittent fever persisted
Day 15	Chest CT: multiple thick-walled cavities (pulmonary necrosis,abscess vs. CPAM not excluded)	Continued antibiotics	Worsening pulmonary necrosis suspected
Day 16–22	Recurrent fever; progressive cavitary lesions on CT; mediastinal shift	Escalated to IV linezolid (10 days)	Fever partially improved; WBC peaked at 28.2 × 10^9/L, then normalized
Day 32	Cough and respiratory distress; CT: left pneumothorax (40–50% compression)	Thoracic drainage; surgery advised but declined by family	Pneumothorax resolved
Day 41	Recurrent fever; nasopharyngeal swab positive for *Mycoplasma pneumoniae*	IV azithromycin (3 days)	Temperature normalized
Day 67	Follow-up CT: decreased number and size of cavities, residual consolidation	Conservative management	Clinical improvement, occasional cough
Month 9	CT: further cavity reduction, fibrous strands, localized bronchiectasis	Supportive care, no recurrence	Patient clinically well
Month 17	CT: minimal residual cavitary lesions, consolidations,stable residual bronchiectasis	Long-term follow-up	Normal growth, no recurrent infections

## Discussion

This case describes a 2-year-old male child with necrotizing pneumonia presenting with persistent fever, respiratory distress, and a prolonged fever lasting 24 days. Laboratory tests revealed leukopenia with nuclear left shift, toxic granules and vacuolation in neutrophils, and elevated D-dimer levels. Chest CT scan showed lung consolidation and cavitation changes. The child was co-infected with *Streptococcus pneumoniae*, influenza virus and human metapneumovirus. The treatment course included the development of pneumothorax, with surgical resection of necrotic lung tissue being declined by the family. Throughout the illness, significant improvement in cavitary lesions was observed, albeit with a prolonged recovery period. These characteristics highlight the severity of the case and the challenges in managing pediatric necrotizing pneumonia comprehensively.

Studies by Masters et al. (2) and Hsieh et al. (4) indicate that necrotizing pneumonia often affects immunocompetent children under 5 years old without underlying health conditions. The subject of this study is a 2-year-old child with no preexisting illnesses. Research findings (2) suggest that even with appropriate treatment, children with necrotizing pneumonia tend to experience severe and prolonged symptoms, including persistent fever and respiratory distress. Similarly, this child exhibited a prolonged fever lasting 24 days and accompanying shortness of breath. Research suggests (2, 5) that infection-induced vasculitis, activation of the coagulation system, and subsequent thrombus formation leading to vascular obstruction and cavity formation may contribute to the development of necrotizing pneumonia. Elevated D-dimer levels and increased platelet count in this child further support this hypothesis.

Studies by Hsieh et al. (3) indicate that heightened IL-8 expression may play a role in the pathogenesis of necrotizing pneumonia. Consistent with these findings, this study observed abnormally high IL-8 levels in the child, along with elevated IL-6 and IL-10 levels. Additionally, research by Kapania and Cavallazzi (6) highlights influenza co-infection as a major risk factor for necrotizing pneumonia. The child in this study tested positive for *Streptococcus pneumoniae*, Influenza A virus, and human metapneumovirus infections. Furthermore, Research (7–11) indicates that influenza virus infection may lead to a reduction in the production of CD3 + CD4 + cells and CD56 + CD16 + cells, while increasing the levels of CD19 + cells and CD3 + CD8 + cells potentially contributing to the development of necrotizing pneumonia. Consistent with previous findings, the patient in this study exhibited decreased levels of CD3 + CD4 + and D56 + CD16 + cells, along with an elevated level of CD19 + cells. However, unlike previous reports, this patient also showed a reduction in CD8 + T cells.

Immunologic evaluation revealed significant abnormalities in this patient. These findings may represent transient immunosuppression secondary to severe infection. However, according to the framework proposed by Moratti et al. (12), an underlying inborn error of immunity (IEI) cannot be definitively excluded without further investigation. Notably, absolute counts of lymphocyte subsets were not performed, and immune parameters were not reassessed during the convalescent phase—both of which limit the ability to rule out IEI. During a follow-up, the child was reported to be in good health without a history of recurrent infections, and the family declined additional immunologic testing. Nevertheless, based on current literature (13), early administration of intravenous immunoglobulin (IVIG) may support recovery in patients with severe infections and concurrent immune abnormalities, particularly when IEI is a potential concern.

Zhang et al. (14) and Ma et al. (15) demonstrated that elevated levels of CRP, WBC, NEUT, and PCT are associated with adverse outcomes in patients with necrotizing pneumonia. In the present case, the child exhibited significantly elevated CRP and PCT levels, consistent with previous findings. However, in an atypical presentation, the child exhibited leukopenia, along with a nuclear left shift, the presence of toxic granules, and vacuolization in neutrophils. These findings strongly indicate the possibility of a bacterial infection when leukopenia is accompanied by these specific indicators.

Furthermore, studies by Ma et al. (15) and Arruda et al. (16) have shown that patients with necrotizing pneumonia often present with anemia, hypoalbuminemia, and hyponatremia. Specifically, Ma et al. (15) reported reductions in both sodium and calcium levels, while Arruda et al. (16) observed only hyponatremia. In this case, the child exhibited anemia and hypoalbuminemia, consistent with previous findings, but sodium levels were within the normal range. Notably, the child demonstrated significantly decreased ionized calcium levels, which may represent an additional metabolic disturbance associated with the disease. This finding highlights the potential role of ionized calcium as a more sensitive indicator of metabolic imbalance in necrotizing pneumonia compared to total calcium levels.

Imaging characteristics play a pivotal role in the diagnosis and monitoring of necrotizing pneumonia. Typical radiologic findings include lung consolidation with low-attenuation areas, solitary or multiple necrotic cavities, air- or fluid-containing cavitations (either thin-walled or lacking clear walls), fibrous strands, emphysema, pneumothorax or hydropneumothorax, and pleural thickening (17). In the present case, the child initially exhibited lung consolidation, which progressed to cavitary lesions by day 15—raising clinical suspicion of necrotizing pneumonia and necessitating differentiation from congenital pulmonary malformations. On day 42, the patient developed a pneumothorax; although surgical removal of necrotic tissue was advised, it was declined by the family. By day 67, cavitary lesions had improved markedly, and further regression was seen by month 9, though bronchiectasis had emerged. By month 17, only minimal residual cavitary lesions and consolidations persisted, along with localized bronchiectasis, without further improvement.

Studies by Mocelin et al. (1) and Bin et al. (18) have indicated that lung lesions in the majority of necrotizing pneumonia cases resolve within 4–6 months. However, in this instance, the resolution of lung lesions extended up to 9 months, aligning with the developmental trajectory of children’s lungs. Given that alveolar development continues until approximately 2–3 years of age (19), and that cavitary resolution was observed by month 9, it is advisable to adopt a conservative, observation-focused approach for 12–18 months to avoid premature surgical intervention. To guide clinical decision-making and monitor complications such as persistent bronchiectasis or recurrent infection, a structured imaging follow-up plan is recommended. This should include low-dose chest CT at key stages, supplemented by chest X-ray or ultrasound to minimize radiation exposure. Suggested intervals are the acute phase (days 15–30), early recovery (2–3 months), 6 months, 9–12 months, and symptom-triggered assessments beyond 12 months. Follow-up should be guided by clinical signs—such as the recurrent fever on day 41—and managed with input from pediatric pulmonology and radiology teams.

The treatment of pediatric necrotizing pneumonia requires a comprehensive approach, including timely antimicrobial therapy, corticosteroids when appropriate bronchoscopy, and surgical intervention. Bronchoalveolar lavage (20) proves beneficial in clearing respiratory secretions, alleviating obstruction, and facilitating lung expansion, particularly in cases of necrotizing pneumonia triggered by *Mycoplasma pneumoniae*. Zhang et al.’s (14, 21) research revealed that children with bacterial necrotizing pneumonia undergo bronchoalveolar lavage less frequently than those with *Mycoplasma pneumoniae* induced necrotizing pneumonia. In the present case, bronchoscopy did not unveil extensive mucus plugs, secretions, necrotic tissue, suggesting caution in selecting bronchoalveolar lavage treatment for bacterial necrotizing pneumonia.

The patient’s lack of pneumococcal and influenza vaccination represents a significant gap in preventive care. The child had not received either the pneumococcal vaccine or the seasonal influenza vaccine, which may have increased the risk of developing severe necrotizing pneumonia. Furthermore, co-infection with multiple pathogens—including *Streptococcus pneumoniae*, influenza A (H3N2), human metapneumovirus, and *Mycoplasma pneumoniae*—placed a substantial burden on the immune system and may have exacerbated immunologic abnormalities, such as reduced percentages of T cells and NK cells, as well as low IgG levels. Administration of pneumococcal vaccination could have mitigated the severity of pneumococcal infection, while influenza vaccination may have reduced the risk of viral-bacterial co-infections, a known driver of necrotizing pneumonia severity (6). Inadequate vaccination may contribute to pathogen overload, further aggravating immune dysregulation. This case highlights a critical deficiency in preventive strategies and underscores the need for catch-up immunization with pneumococcal vaccination, along with annual influenza vaccination. These measures, combined with appropriate immunologic evaluation, are essential for reducing the risk of future infections and identifying potential underlying immunodeficiencies.

The patient’s parents reflected on their experience throughout the illness. Although surgical intervention was advised, they decided against it, believing that necrotizing pneumonia might resolve spontaneously over 2–3 years. They also associated the onset of necrotizing pneumonia with the bronchoscopy procedure, but acknowledged that this could represent part of the natural disease course rather than a direct cause. The parents emphasized that they understood the medical team’s intentions, recognizing that all treatments were aimed at the child’s well-being. They further noted that both families and physicians face inherent limitations in fully grasping the evolving condition, describing the process as one of shared uncertainty and decision-making under complexity.

## Conclusion

This case highlights the complex interplay between severe necrotizing pneumonia, immunologic abnormalities, co-infection, and gaps in vaccination in a young child. The prolonged disease course, coupled with evolving imaging features and immune dysregulation, underscores the importance of comprehensive diagnostic evaluation, including immunologic work-up and long-term radiologic follow-up. Although the patient ultimately recovered without surgical intervention, the initial concern for congenital lung malformation and persistent bronchiectasis illustrate the need for cautious, individualized clinical decision-making. Timely vaccination, early immunologic assessment, and multidisciplinary management are critical to optimizing outcomes and preventing recurrence in similar pediatric cases. This report also reinforces the value of conservative observation in young children, given ongoing lung development, and supports structured imaging surveillance to guide treatment strategies.

## Data Availability

The original contributions presented in the study are included in the article/supplementary material, further inquiries can be directed to the corresponding author.
